# Optimal model for 4-band biorthogonal wavelets bases for fast calculation

**DOI:** 10.1186/s13660-017-1495-8

**Published:** 2017-09-13

**Authors:** Qingyun Zou, Guoqiu Wang

**Affiliations:** 1 Hunan Province Cooperative Innovation Center for The Construction Development of Dongting Lake Ecological Economic Zone, Hunan University of Arts and Science, Changde, Hunan 415000 P.R. China; 2 College of Mathematics and Computer Science, Hunan Normal University, Changsha, Hunan 410081 P.R. China

**Keywords:** 42C40, 42A16, multi-band wavelets, circular matrix, biorthogonal wavelets, vanishing movement, symmetry

## Abstract

A class of 4-band symmetric biorthogonal wavelet bases has been constructed, in which any wavelet system the high-pass filters can be determined by exchanging position and changing the sign of the two low-pass filters. Thus, the least restrictive conditions are needed for forming a wavelet so that the free degrees can be reversed for application requirement. Some concrete examples with high vanishing moments are also given, the properties of the transformation matrix are studied and the optimal model is constructed. These wavelets can process the boundary conveniently, and they lead to highly efficient computations in applications.

## Introduction

As a generalization of orthogonal wavelets, the biorthogonal wavelets have become a fundamental tool in many areas of applied mathematics, from signal processing to numerical analysis [1–8]. It is well known that 2-band orthogonal wavelets, suffering from severe constraint conditions, such as nontrivial symmetric 2-band orthogonal wavelets, do not exist [9]. Biorthogonal wavelets, multi-band wavelets are designed as alternatives for more freedom and flexibility [10–15]. Multi-band wavelets have attracted considerable attention due to their richer parameter space to have a more flexible time-frequency tiling, to zoom in onto narrow band high frequency components in frequency responses, to give better energy compaction than 2-band wavelets. (Bi)orthogonal real-valued multi-band wavelets with symmetry have been reported in [10–12, 16, 17]. In this paper, we can construct innumerable wavelet filters with some structure for fast calculation, among which we can select the best ones for practical applications.

As the case of a dyadic wavelet, a pair $(\phi(x),\widetilde{\phi}(x))$ of dual scaling functions can be expressed as the following dilation equations:

1.1 $$ \phi(x)=\sum_{k\in Z}h_{k} \phi(dx-k)$$

and

1.2 $$ \widetilde{\phi}(x)=\sum_{k\in Z} \widetilde{h}_{k}\widetilde{\phi}(dx-k),$$

respectively, with

1.3 $$ \bigl\langle \phi(\cdot-k),\widetilde{\phi}(\cdot-j) \bigr\rangle = \delta_{k-j} $$

or equivalently,

1.4 $$ \sum_{k} h_{k} \widetilde{h}_{k+dj}=\delta_{j}, $$

where $\delta_{j}$ denotes the *Dirac* sequence such that $\delta_{j}=1 $ for $j=0$ otherwise $\delta_{j}=0 $.

Recall the sub-band coding scheme or Mallat algorithm associated to a 4-band biorthogonal wavelets. There are eight sequences $h=(h_{n})_{n\in Z}$, $g^{i}=(g^{i}_{n})_{n\in Z}$ ($i=1,2,3$), $\widetilde{h}=(\widetilde{h}_{n})_{n\in Z}$, $\widetilde{g^{i}}=(\widetilde{g}^{i}_{n})_{n\in Z}$ ($i=1,2,3$), four of which are used for decomposition $\{h,g^{1},g^{2},g^{3}\}$ and the others for reconstruction $\{\widetilde{h}, \widetilde{g}^{1},\widetilde{g}^{2},\widetilde{g}^{3}\}$. Starting from a data sequence $x=(x_{n})_{n\in Z}$, we convolve with *h*, $g^{1}$, $g^{2}$, $g^{3}$ and retain only one sample out of every four for decomposition

1.5 $$ \begin{gathered} c_{n}=\sum _{k}h_{4n-k}x_{k}, \\ d^{i}_{n}=\sum_{k}g^{i}_{4n-k}x_{k},\quad i=1,2,3. \end{gathered} $$

The reconstruction operation is

1.6 $$ x_{k}=\sum_{n}\Biggl( \widetilde{h}_{4n-k}c_{n} + \sum^{3}_{i=1} \widetilde{g}^{i}_{4n-k}d^{i}_{n}\Biggr). $$

Equations (1.5) and (1.6) can be rewritten as the form of 4-circular matrix [16], which can be defined by the 4-circular operator.

If $c^{0}$ is a periodic signal, we rewrite $c^{0}=(c^{0}_{1},c^{0}_{2},\ldots,c^{0}_{4n})^{T}$, which is a whole period.

Let $c^{1}=(c^{1}_{1},c^{1}_{2},\ldots,c^{1}_{n},d^{1}_{1},d^{1}_{2},\ldots ,d^{1}_{n},d^{2}_{1},d^{2}_{2},\ldots,d^{2}_{n},d^{3}_{1},d^{3}_{2},\ldots,d^{3}_{n})^{T}$. Then there exists a $4n\times4n$ 4-circular matrix $M_{4n}$ generated by $\{h,g^{1},g^{2},g^{3}\}$ such that

1.7 $$ c^{1}=M_{4n}c^{0}. $$

It is easy to see that (1.7) is equivalent to (1.5). Let

1.8 $$ c^{0}=\widetilde{M}_{4n}^{T}c^{1}, $$

where $\widetilde{M}_{4n}$ is a 4-circular $4n\times4n$ matrix generated by $\{\widetilde{h},\widetilde{g}^{1},\widetilde{g}^{2},\widetilde{g}^{3}\}$.

Clearly, (1.8) is equivalent to (1.6). Then

1.9 $$ \big\| c^{1}\big\| ^{2}=\bigl(c^{1}\bigr)^{T}c^{1}= \bigl(c^{0}\bigr)^{T}\bigl(M^{T}_{4n} M_{4n}\bigr)c^{0}. $$

Since $M^{T}_{4n} M_{4n}$ is a positive definite matrix, its eigenvalues $\lambda_{i}$ ($i=1,2\ldots,4n$) are positive and there exists an orthonormal matrix *Q* such that

1.10 $$ M^{T}_{4n}M_{4n}=Q^{T} \operatorname{diag}( \lambda_{1},\lambda_{2},\ldots ,\lambda_{4n})Q. $$

Let $s=Qc^{0}=(s_{1},s_{2},\ldots,s_{4n})^{T}$. Then $\|s\|^{2}=\|c^{0}\|^{2}$. It follows from (1.9) and (1.10) that

$$\big\| c^{1}\big\| ^{2}=\bigl(Qc^{0}\bigr)^{T} \operatorname{diag}(\lambda_{1},\lambda_{2},\ldots,\lambda _{4n}) \bigl(Qc^{0}\bigr)=\sum^{4n}_{i=1} \lambda_{i}s_{i}^{2}. $$

Thus,

1.11 $$ \min\{\lambda_{i}\}\big\| c^{0}\big\| ^{2}\leq \big\| c^{1}\big\| ^{2}\leq\max\{ \lambda_{i}\} \big\| c^{0}\big\| ^{2}. $$

Similarly,

1.12 $$ \min\{\widetilde{\lambda}_{i}\}\|c^{0}\|^{2}\leq \|c^{1}\| ^{2}\leq\max\{\widetilde{\lambda}_{i}\} \|c^{0}\|^{2}, $$

where $\widetilde{\lambda}_{i}$ ($i=1,2\ldots,4n$) are the eigenvalues of $\widetilde{M}^{T}_{4n} \widetilde{M}_{4n}$.

We try to calculate the eigenvalues of $M^{T}_{4n} M_{4n}$ or $\widetilde{M}^{T}_{4n} \widetilde{M}_{4n}$. It has been shown that the eigenvalues of $M^{T}_{4n} M_{4n}$ appear in pairs of reciprocals, $M^{T}_{4n} M_{4n}$ and $\widetilde{M}^{T}_{4n} \widetilde{M}_{4n}$ have the same eigenvalues. It is obvious that $\max\{\lambda_{i}\}=\max\{\widetilde{\lambda}_{i}\}=\frac{1}{\min\{\lambda _{i}\}}=\frac{1}{\min\{\widetilde{\lambda}_{i}\}} $.

This paper is organized as follows. In Section 2, we design a type of 4-band biorthogonal wavelets filters for fast calculation, give the construction method. In Section 3, some results of the wavelet transform matrix are developed. So a model to minimize the maximum eigenvalue is built in Section 4. An example is provided to illustrate our results in this paper. Conclusions and future work are summarized in Section 5.

## 4-band biorthogonal wavelets filters for fast calculation

In this section, a class of 4-band symmetric biorthogonal wavelet filters for fast calculation is designed, and the corresponding wavelet filters are constructed.

Assume that

2.1 $$\begin{aligned}& h=\{t_{0},t_{1},\ldots, t_{2L-1},t_{2L-1}, \ldots, t_{1},t_{0}\}, \end{aligned}$$

2.2 $$\begin{aligned}& \widetilde{h}=\{\widetilde{t}_{0},\widetilde{t}_{1},\ldots, \widetilde {t}_{2L-1},\widetilde{t}_{2L-1},\ldots, \widetilde{t}_{1},\widetilde{t}_{0}\}. \end{aligned}$$

The high-pass filters for decomposition are

2.3 $$ \begin{gathered} g^{1}_{2i}=(-1)^{L-i}h_{2i+1},\qquad g^{1}_{2i+1}=(-1)^{L-i+1}h_{2i}\quad (0\leq i \leq2L-1), \\ g^{2}_{i}=(-1)^{i}\widetilde{h}_{4L-1-i}\quad (0 \leq i \leq4L-1), \\ g^{3}_{i}=(-1)^{i}\widetilde{g}^{1}_{4L-1-i}\quad (0\leq i \leq4L-1). \end{gathered} $$

Note that $g^{1}$ is symmetric and $g^{2}$ and $g^{3}$ are antisymmetric.

The high-pass filters for reconstruction are

2.4 $$ \begin{gathered} \widetilde{g}^{1}_{2i}=(-1)^{L-i} \widetilde{h}_{2i+1}, \quad\quad\widetilde {g}^{1}_{2i+1}=(-1)^{L-i+1} \widetilde{h}_{2i} \quad (0\leq i \leq2L-1), \\ \widetilde{g}^{2}_{i}=(-1)^{i}h_{4L-1-i} \quad(0 \leq i \leq4L-1), \\ \widetilde{g}^{3}_{i}=(-1)^{i}g^{1}_{4L-1-i}\quad (0\leq i \leq4L-1). \end{gathered} $$

We can see that $\widetilde{g}^{1}$ is symmetric and $\widetilde{g}^{2}$ and $\widetilde{g}^{3}$ are antisymmetric.

For $L = 3$, an example of a filter bank $\{h,g^{1}, g^{2}, g^{3} \}$ is as follows:

$$\left [ \textstyle\begin{array}{c@{\quad}c@{\quad}c@{\quad}c@{\quad}c@{\quad}c@{\quad}c@{\quad}c@{\quad}c@{\quad}c@{\quad}c@{\quad}c} t_{0} & t_{1} & t_{2} & t_{3} & t_{4} & t_{5} & t_{5} & t_{4} & t_{3} & t_{2} & t_{1} & t_{0} \\ t_{1} & -t_{0} & -t_{3} & t_{2} & t_{5} & -t_{4} & -t_{4} & t_{5} & t_{2} & -t_{3} & -t_{0} & t_{1} \\ \widetilde{t}_{0} & -\widetilde{t}_{1} &\widetilde{t}_{2} & -\widetilde{t}_{3} & \widetilde{t}_{4}& -\widetilde{t}_{5} & \widetilde{t}_{5} & -\widetilde{t}_{4} & \widetilde{t}_{3} & -\widetilde{t}_{2} &\widetilde{t}_{1} & -\widetilde {t}_{0} \\ \widetilde{t}_{1} & \widetilde{t}_{0} & -\widetilde{t}_{3} & -\widetilde {t}_{2} & \widetilde{t}_{5}& \widetilde{t}_{4} & -\widetilde{t}_{4} & -\widetilde{t}_{5} & \widetilde{t}_{2} & \widetilde{t}_{3} & -\widetilde{t}_{0} & -\widetilde{t}_{1} \end{array}\displaystyle \right ]. $$

Clearly, this type of wavelet filter banks are only determined the two low-pass filters *h*, *h̃*, *i.e.*, the high-pass filters can be determined by exchanging position and changing the sign of the two low-pass filters. Therefore, it can reduce the computational complexity and facilitate fast computation.

In addition, we assume $\sum_{k}h_{k}=\sum_{k}\widetilde{h}_{k}=2 $. At this time, the basic conditions of biorthogonal wavelets are

$$\begin{gathered} \sum_{k} h_{k} \widetilde{h}_{k+4j}=\delta_{j}, \\ \sum_{k} h_{k}\widetilde{g}^{n}_{k+4j}=0,\quad n=1,2, 3, \\ \sum_{k} g^{n}_{k} \widetilde{g}^{m}_{k+4j}=\delta_{n-m} \delta_{j},\quad 1\leq n,m\leq 3. \end{gathered} $$

We have the following theorem for the biorthogonality condition.

### Theorem 2.1

*If*

2.5 $$ \begin{gathered} \sum_{k} h_{k} \widetilde{h}_{k+4j}=\delta_{j}, \\ \sum_{k} {g}^{1}_{k} \widetilde{h}_{k+4j}=0, \end{gathered} $$

*then the wavelet filter banks defined by* (2.1)-(2.4) *satisfy the biorthogonality condition*.

### Proof

By direct calculation, we can see that $\sum_{k} h_{k}\widetilde{g}^{n}_{k+4j}=0$ ($n=2,3$), $\sum_{k} g^{1}_{k}\widetilde{g}^{n}_{k+4j}=0$ (${n=2,3}$), $\sum_{k} g^{n}_{k}\widetilde{h}_{k+4j}=0$ ($n=2,3$), $\sum_{k} g^{n}_{k}\widetilde{g}^{1}_{k+4j}=0$ ($n=2,3$).

From $\sum_{k} h_{k} \widetilde{h}_{k+4j}=\delta_{j}$, we can obtain $\sum_{k} g^{n}_{k}\widetilde{g}^{n}_{k+4j}=\delta_{j}$ ($n=1,2,3$). From $\sum_{k} g^{1}_{k}\widetilde{h}_{k+4j}=0$, we can obtain $\sum_{k} h_{k}\widetilde{g}^{1}_{k+4j}=0 $, $\sum_{k} g^{2}_{k}\widetilde{g}^{3}_{k+4j}=0$, $\sum_{k} g^{3}_{k}\widetilde{g}^{2}_{k+4j}=0$. The proof is complete. □

### Remark 2.1

According to Theorem 2.1, for 4*L* parameters in the wavelet system, (2.5) contains 2*L* nonlinear equations. We can add constraints such as high vanishing movements for the surplus 2*L* parameters.

### Example 2.1

We relax the condition of the sum of highest vanishing moments, and let ${L=3}$ and the high-pass wavelet filters defined by (2.3) have 3, 4, 3 order vanishing moments, respectively. The parameterized filters are as follows:

$$\begin{gathered} t_{0}=\frac{-1}{10}\frac{49\mbox{,}152x^{3}-25\mbox{,}088x^{2}+4\mbox{,}120x-219}{2\mbox{,}048x^{2}-960x+113},\\ t_{1}=\frac{-1}{40}\frac{65\mbox{,}536x^{3}-36\mbox{,}864x^{2}+6\mbox{,}240x-307}{2\mbox{,}048x^{2}-960x+113}, \\ t_{2}=\frac{1}{40}\frac{65\mbox{,}536x^{3}-16\mbox{,}384x^{2}-800x+183}{2\mbox{,}048x^{2}-960x+113}, \\ t_{3}=\frac{1}{ 20}\frac{98\mbox{,}304x^{3}-39\mbox{,}936x^{2}+4\mbox{,}720x-153}{2\mbox{,}048x^{2}-960x+113}, \\ t_{4}=\frac{1}{4}\frac{2\mbox{,}048x^{2}-1\mbox{,}216x+169}{2\mbox{,}048x^{2}-960x+113}, \\ t_{5}=\frac{1}{4}\frac{2\mbox{,}048x^{2}-1\mbox{,}216x+177}{2\mbox{,}048x^{2}-960x+113}, \\ \widetilde{t}_{0}=x-\frac{3}{16},\\\widetilde{t}_{1}=x- \frac{5}{32}, \\ \widetilde{t}_{2}=x-\frac{3}{32},\\\widetilde{t}_{3}=x , \\ \widetilde{t}_{4}=-2x+\frac{11}{16}, \\ \widetilde{t}_{5}=-2x+\frac{3}{4}, \end{gathered} $$

where *x* is a free parameter.

## The properties of the wavelet transformation matrix

A so-called 4-circular matrix [8], which is generated by the filters *h*, $g^{1}$, $g^{2}$, $g^{3}$, is denoted as $M_{4n} $. For $n=3$, $M_{12} $ is as follows:

$$M_{12}=\left [ \textstyle\begin{array}{l@{\quad}l@{\quad}l@{\quad}l@{\quad}l@{\quad}l@{\quad}l@{\quad}l@{\quad}l@{\quad}l@{\quad}l@{\quad}l} h_{0} & h_{1} & h_{2} & h_{3} & 0 & 0 & 0 &0 &h_{-4} & h_{-3} & h_{-2} & h_{-1}\\ h_{-4} & h_{-3} &h_{-2} & h_{-1} & h_{0} & h_{1}&h_{2} & h_{3}& 0 & 0& 0 & 0\\ 0 & 0 & 0 & 0 & h_{-4} & h_{-3} &h_{-2} & h_{-1} & h_{0} & h_{1} & h_{2} & h_{3} \\ g^{1}_{0} & g^{1}_{1} & g^{1}_{2} & g^{1}_{3} & 0 & 0 & 0 & 0& g^{1}_{-4} & g^{1}_{-3} & g^{1}_{-2} & g^{1}_{-1}\\ g^{1}_{-4} & g^{1}_{-3} & g^{1}_{-2} & g^{1}_{-1}& g^{1}_{0} & g^{1}_{1} & g^{1}_{2} & g^{1}_{3}& 0 & 0 & 0 & 0\\ 0 & 0 & 0 & 0 & g^{1}_{-4} & g^{1}_{-3} & g^{1}_{-2} & g^{1}_{-1}& g^{1}_{0} & g^{1}_{1} & g^{1}_{2} & g^{1}_{3}\\ g^{2}_{0} & g^{2}_{1} & g^{2}_{2} & g^{2}_{3} & 0 & 0 & 0 & 0& g^{2}_{-4} & g^{2}_{-3} &g^{2}_{-2} & g^{2}_{-1}\\ g^{2}_{-4} & g^{2}_{-3} & g^{2}_{-2} & g^{2}_{-1}& g^{2}_{0} & g^{2}_{1} & g^{2}_{2} & g^{2}_{3} & 0 & 0 & 0 & 0\\ 0 & 0 & 0 & 0 & g^{2}_{-4} & g^{2}_{-3} & g^{2}_{-2} & g^{2}_{-1}& g^{2}_{0} & g^{2}_{1} & g^{2}_{2} & g^{2}_{3}\\ g^{3}_{0} & g^{3}_{1} & g^{3}_{2} & g^{3}_{3} & 0 & 0 & 0 & 0&g^{3}_{-4}& g^{3}_{-3} & g^{3}_{-2} &g^{3}_{-1}\\ g^{3}_{-4} & g^{3}_{-3} & g^{3}_{-2} & g^{3}_{-1}& g^{3}_{0} & g^{3}_{1} & g^{3}_{2} & g^{3}_{3} & 0 & 0 & 0 & 0\\ 0 & 0 & 0 & 0& g^{3}_{-4} & g^{3}_{-3} & g^{3}_{-2} & g^{3}_{-1}& g^{3}_{0} & g^{3}_{1} & g^{3}_{2} & g^{3}_{3} \end{array}\displaystyle \right ]. $$

Define

3.1 $$ \begin{gathered} MM^{T}=\left [ \textstyle\begin{array}{c@{\quad}c@{\quad}c@{\quad}c} HH^{T} & HG_{1}^{T} & HG_{2}^{T} & HG_{3}^{T} \\ G_{1}H^{T} & G_{1}G_{1}^{T} & G_{1}G_{2}^{T} & G_{1}G_{3}^{T} \\ G_{2}H^{T} & G_{2}G_{1}^{T} & G_{2}G_{2}^{T} & G_{2}G_{3}^{T} \\ G_{3}H^{T} & G_{3}G_{1}^{T} & G_{3}G_{2}^{T} & G_{3}G_{3}^{T} \end{array}\displaystyle \right ], \\ \widetilde{M}\widetilde{M}^{T}=\left [ \textstyle\begin{array}{c@{\quad}c@{\quad}c@{\quad}c} \widetilde{H}\widetilde{H}^{T} & \widetilde{H}\widetilde{G}_{1}^{T} & \widetilde{H}\widetilde{G}_{2}^{T} & \widetilde{H}\widetilde{G}_{3}^{T} \\ \widetilde{G}_{1}\widetilde{H}^{T} & \widetilde{ G}_{1}\widetilde{G}_{1}^{T} & \widetilde{G}_{1}\widetilde{G}_{2}^{T} & \widetilde{G}_{1}\widetilde{G}_{3}^{T} \\ \widetilde{G}_{2}\widetilde{H}^{T} & \widetilde{G}_{2}\widetilde{G}_{1}^{T} & \widetilde{G}_{2}\widetilde{G}_{2}^{T} & \widetilde{G}_{2}\widetilde{G}_{3}^{T} \\ \widetilde{G}_{3}\widetilde{H}^{T} & \widetilde{G}_{3}\widetilde{G}_{1}^{T} & \widetilde{G}_{3}\widetilde{G}_{2}^{T} & \widetilde{G}_{3}\widetilde{G}_{3}^{T} \end{array}\displaystyle \right ], \end{gathered} $$

where *H*, $G_{1}$, $G_{2}$, $G_{3}$ and *H̃*, $\widetilde{G}_{1}$, $\widetilde{G}_{2}$, $\widetilde{G}_{3}$ are all $n\times4n$ 4-circular matrices generated by $\{h,g^{1},g^{2},g^{3}\}$, $\{\widetilde{h},\widetilde{g}^{1},\widetilde{g}^{2},\widetilde{g}^{3}\}$, respectively.

### Theorem 3.1

*Assume that the type of wavelet filter banks defined by* (2.1)-(2.4) *satisfy* (2.5). *Then*, *for any large enough integer* *n*, $M_{n}M_{n}^{T}$ *and* $\widetilde{M}_{n}\widetilde{M}_{n}^{T}$ *defined by* (3.1) *are similar matrices*.

### Proof

The element at the *j*th row and the *k*th column in $G_{1}G_{1}^{T}$ can be written as

$$\begin{aligned} \sum_{i}g^{1}_{i+4j}g^{1}_{i+4k} ={}&\sum_{i\in2Z}(-1)^{(i+4j)/2}h_{(i+4j)+1}(-1)^{(i+4k)/2}h_{(i+4k)+1} \\ &+\sum_{i\in 2Z+1}(-1)^{(4L+1-(i+4j-1)/2)}h_{(i+4j)-1}(-1)^{(4L+1-(i+4k-1)/2)}h_{(i+4k)-1} \\ ={}&\sum_{i\in2Z}h_{i+4j+1}h_{i+4k+1} +\sum _{i\in 2Z+1}h_{i+4j-1}h_{i+4k-1} \\ ={}&\sum_{i}h_{i+4j}h_{i+4k}. \end{aligned} $$

It is just the element in $HH^{T}$ at the same position. Therefore,

$$\begin{gathered} HG_{1}^{T}=G_{1}H^{T}=G_{2}G_{3}^{T}=G_{3}G_{2}^{T}=0,\\HG_{2}^{T}=G_{3}G_{1}^{T}=-G_{2}H^{T}=-G_{1}G_{3}^{T}, \\ HG_{3}^{T}=G_{1}G_{2}^{T}=-G_{2}G_{1}^{T}=-G_{3}H^{T},\\G_{2}G_{2}^{T}=G_{3}G_{3}^{T}. \end{gathered} $$

Similarly, we can show that

$$\begin{gathered} \widetilde{G}_{1}\widetilde{G}_{1}^{T}= \widetilde{H}\widetilde{H}^{T},\\ \widetilde{G}_{2} \widetilde{G}_{2}^{T}=\widetilde{G}_{3} \widetilde{G}_{3}^{T}, \\\widetilde{H}\widetilde{G}_{2}^{T}= \widetilde{G}_{3}\widetilde {G}_{1}^{T}=- \widetilde{G}_{2}\widetilde{H}^{T}=-\widetilde{G}_{1} \widetilde {G}_{3}^{T}, \\ \widetilde{H}\widetilde{G}_{3}^{T}=\widetilde{G}_{1} \widetilde {G}_{2}^{T}=-\widetilde{G}_{2} \widetilde{G}_{1}^{T}=-\widetilde{G}_{3} \widetilde{H}^{T}, \\\widetilde{H}\widetilde{G}_{1}^{T}= \widetilde{G}_{1}\widetilde{H}^{T}=\widetilde {G}_{2}\widetilde{G}_{3}^{T}=\widetilde{G}_{3} \widetilde{G}_{2}^{T}=0. \end{gathered} $$

The element at the *j*th row and the *k*th column in $G_{2}G_{2}^{T}$ can be written as

$$\begin{aligned} \sum_{i}g^{2}_{i+4j}g^{2}_{i+4k} &=\sum_{i}(-1)^{(i+4j)}\widetilde{h}_{4L-(i+4j)-1}(-1)^{(i+4k)} \widetilde {h}_{4L-(i+4k)-1} \\ &=\sum_{i}\widetilde{h}_{4L-(i+4j)-1} \widetilde{h}_{4L-(i+4k)-1} \\ &=\sum_{i}\widetilde{h}_{-i+4j} \widetilde{h}_{-i+4k} \\ &=\sum_{i}\widetilde{h}_{i+4j} \widetilde{h}_{i+4k}. \end{aligned} $$

It is just the element in $\widetilde{H}\widetilde{H}^{T}$ at the same position.

Therefore,

$$\widetilde{G}_{2}\widetilde{G}_{2}^{T}=HH^{T},\qquad HG_{2}^{T}=-\widetilde{H}\widetilde {G}_{2}^{T},\qquad HG_{3}^{T}=-\widetilde{H}\widetilde{G}_{3}^{T}. $$

Define

$$B=\left [ \textstyle\begin{array}{c@{\quad}c@{\quad}c@{\quad}c} O & O & O & I \\ O & O & -I & O \\ O & -I & O & O \\ I & O & O & O \end{array}\displaystyle \right ],\qquad B^{-1}=\left [ \textstyle\begin{array}{c@{\quad}c@{\quad}c@{\quad}c} O & O & O & I \\ O & O & -I & O \\ O & -I & O & O \\ I & O & O & O \end{array}\displaystyle \right ], $$

where $I_{n}$, $O_{n}$ are $n\times n$ identity matrix and zero matrix, respectively, for convenience, we omit the subscript $n\times n$ when it does not cause any confusion.

We can see that $B_{4n}^{-1}(M_{n}M_{n}^{T})B_{4n}=\widetilde{M}_{n}\widetilde{M}_{n}^{T}$. It implies that $M_{n}M_{n}^{T}$ and $\widetilde{M}_{n}\widetilde{M}_{n}^{T}$ are similar matrices. □

### Corollary 3.1

*If* *λ* *is an eigenvalue of matrix* $M_{n}M_{n}^{T}$, *then* $1/\lambda$ *is also an eigenvalue of matrix* $M_{n}M_{n}^{T}$.

### Proof

Note that $M_{n}{M_{n}^{T}}(\widetilde{M}_{ n}\widetilde{M}_{n}^{T})=I_{4n}$. It implies that both $M_{n}M_{n}^{T}$ and $\widetilde{M}_{n}\widetilde{M}_{n}^{T}$ are positive definite matrices, and all of eigenvalues of $M_{n}M_{n}^{T}$ and $\widetilde{M}_{n}\widetilde{M}_{n}^{T}$ are positive. It follows from Theorem 3.1 that $M_{n}M_{n}^{T}$ and $\widetilde{M}_{n}\widetilde{M}_{n}^{T}$ have the same eigenvalues. The proof is completed. □

Now we can state the lower bound theorem as follows.

### Theorem 3.2

3.2 $$ \max_{1\leq i\leq{4n}}\lambda_{i} \geq \max \biggl\{ \frac{\sum_{i}h^{2}_{i}+\sum_{i}\widetilde{h}_{i}^{2}}{2} ,\frac{2}{\sum_{i}h^{2}_{i}+\sum_{i}\widetilde{h}_{i}^{2}} \biggr\} . $$

### Proof

Assume that the type of wavelet filter banks defined by (2.1)-(2.4) satisfy (2.5). The elements at the diagonal of matrix $M_{4n}M_{4n}^{T}$ are $\sum_{i}h^{2}_{i}$ or $\sum_{i}g^{2}_{i}$.

Thus,

$$\sum_{i=1}^{4n}\lambda_{i} =2n \biggl(\sum_{i}h^{2}_{i}+\sum _{i}\widetilde{h}^{2}_{i}\biggr). $$

According to Corollary 3.1, we have

$$\max_{i}\lambda_{i} \geq\frac{\sum_{i=1}^{4n}\lambda_{i} }{4n}\geq\min _{i}\lambda_{i} =\frac{1}{\max_{i}\lambda_{i} } , $$

this leads to

$$\max_{1\leq i\leq{4n}}\lambda_{i} \geq\max \biggl\{ \frac{\sum_{i}h^{2}_{i}+\sum_{i}\widetilde{h}_{i}^{2}}{2},\frac{2}{\sum_{i}h^{2}_{i}+\sum_{i}\widetilde{h}_{i}^{2}} \biggr\} . $$

The proof is complete. □

### Remark 3.1

Theorem 3.2 shows that there exists a lower bound on the maximum eigenvalue of the wavelet transform matrices which is only related to the filters and independent of the dimension of the transformation matrix.

## Optimal model for 4-band biorthogonal wavelets bases

In the former section, it has been shown that the eigenvalues of $M^{T}_{4n} M_{4n}$ appear in pairs of reciprocal, $M^{T}_{4n} M_{4n}$ and $\widetilde{M}^{T}_{4n} \widetilde{M}_{4n}$ have the same eigenvalues. It is obvious that $\max\{\lambda_{i}\}=\max\{\widetilde{\lambda}_{i}\}=\frac{1}{\min\{\lambda _{i}\}}=\frac{1}{\min\{\widetilde{\lambda}_{i}\}} $. In this section, we shall design wavelets based on minimizing the maximum eigenvalue.

*Optimal model*. Assume that the type of wavelet filter banks defined by (2.1)-(2.4) with parameter set Ω and $\lambda(\omega)$ are the eigenvalues of $M^{T}_{4n} M_{4n}$. The optimal model for 4-band biorthogonal wavelet bases is

4.1 $$ \min_{\omega\in\Omega}\max_{1\leq i\leq{4n}}\lambda_{i}( \omega). $$

### Example 4.1

For $L=3$, the parameterized filters have been given in Example 2.1. We can use an extra degree of freedom to minimize the maximum eigenvalue. From (4.1), we can obtain the solution is $x\approx0.11097$. The wavelets are denoted as Op(12-12). At this time,

$$\begin{gathered} t_{0}=0.01129264,\qquad t_{1}=-0.01660958 ,\qquad t_{2}= -0.01418315,\\ t_{3}= 0.02102888 ,\qquad t_{4}=0.4676785,\qquad t_{5}=0.5307927,\\ \widetilde{t}_{0}= -0.07653 ,\quad\quad\widetilde{t}_{1}=-0.04528, \quad\quad\widetilde{t}_{2}=0.01722 ,\quad\quad \widetilde{t}_{3}= 0.11097, \\\widetilde{t}_{4}= 0.46556,\quad\quad \widetilde{t}_{5}= 0.52806. \end{gathered} $$

**Figure 1:**
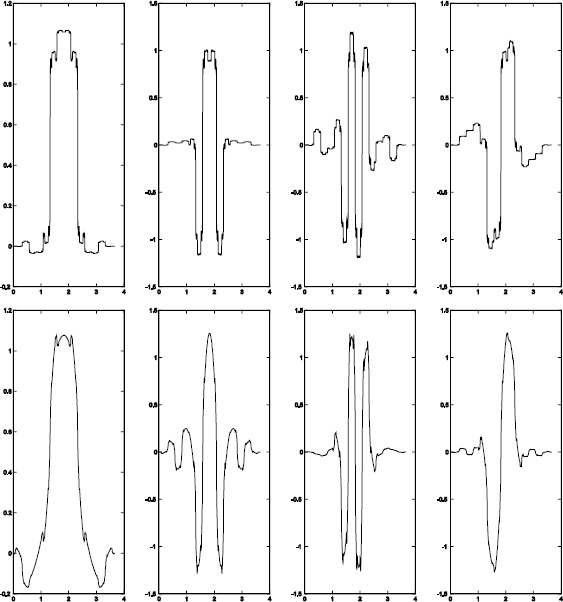
**The graphs of Op(12-12) in Example** **4.1** **.**

See Figure 1 for the graphs of the scaling functions and wavelets in this example. We have

$$\begin{aligned} \lambda(M_{20})={}&\{0.7775, 0.7775, 0.7775, 0.7775, 0.8555, 0.8555, 0.8555, 0.8555, 1, 1, \\ &1, 1, 1.1689, 1.1689, 1.1689, 1.1689, 1.2863, 1.2863,1.2863, 1.2863\}. \end{aligned}$$

Note that $x=1/9$; very near 0.11097. We can obtain the wavelets with rational filter banks as follows:

$$\begin{gathered}t_{0}=857/76\mbox{,}830,\qquad t_{1}= -4\mbox{,}813\mbox{,}531\mbox{,}065\mbox{,}998\mbox{,}413/288\mbox{,}230\mbox{,}376\mbox{,}151\mbox{,}711\mbox{,}744 ,\qquad \\ t_{2}= -2\mbox{,}081\mbox{,}007\mbox{,}309\mbox{,}269\mbox{,}241/144\mbox{,}115\mbox{,}188\mbox{,}075\mbox{,}855\mbox{,}872,\\ t_{3}= 3\mbox{,}020\mbox{,}922\mbox{,}951\mbox{,}921\mbox{,}967/144\mbox{,}115\mbox{,}188\mbox{,}075\mbox{,}855\mbox{,}872, \\ t_{4}= 4\mbox{,}793/10\mbox{,}244,\qquad t_{5}=5\mbox{,}441/10\mbox{,}244,\\ \widetilde{t}_{0}=-11/144 ,\qquad\widetilde{t}_{1}=-13/288 , \qquad\widetilde{t}_{2}=5/288,\qquad \widetilde{t}_{3}= 1/9,\\ \widetilde{t}_{4}= 67/144, \qquad\widetilde{t}_{5}= 19/36. \end{gathered} $$

## Conclusions and future work

We have constructed a class of 4-band symmetric biorthogonal wavelet bases, in which any wavelet system the high-pass filters can be determined by exchanging position and changing the sign of the two low-pass filters. The transformation matrix is studied and the optimal model is constructed. A concrete example with high vanishing moments is also given which leads to highly efficient computations. We will further study the related topic that this wavelet bases are applied in numerical calculation and image compression coding.
